# Guarana (*Paullinia cupana*) as a potential tool for mesenchymal stromal cells priming in regenerative medicine

**DOI:** 10.1590/1414-431X2024e13286

**Published:** 2024-07-29

**Authors:** D.H. Sirena, A.B. Araújo, A.B.T da Silveira, M.A. Serafini, M.M.F. da Silva, A.K. Silveira, E. Filippi-Chiela, J.C.F. Moreira, A.H. Paz

**Affiliations:** 1 Laboratório de Células, Tecidos e Genes - Centro de Pesquisa Experimental (CPE), Hospital de Clínicas de Porto Alegre, Porto Alegre, RS, Brasil; 2 Programa de Pós Graduação em Fisiologia, Instituto de Ciências Básicas da Saúde, Universidade Federal do Rio Grande do Sul, Porto Alegre, RS, Brasil; 3 Centro de Processamento Celular, Serviço de Hemoterapia, Hospital de Clínicas de Porto Alegre, Porto Alegre, RS, Brasil; 4 Departamento de Bioquímica, Universidade Federal do Rio Grande do Sul, Porto Alegre, RS, Brasil; 5 Departamento de Ciências Morfológicas, Instituto de Ciências Básicas da Saúde, Universidade Federal do Rio Grande do Sul, Porto Alegre, RS, Brasil

**Keywords:** Guarana extract, Paullinia cupana, Mesenchymal stromal cells, Cell therapy, Priming, Caffeine

## Abstract

Mesenchymal stromal cells (MSCs) have therapeutic potential due to their abilities of differentiation, immunomodulation, and migration to injured tissues, potentiating such effects when cells are activated. Guarana (*Paullinia cupana*) is a tropical plant species found in South America that is known for its antioxidant, stimulant, and cicatricial effects. The guarana extract is composed of many substances and caffeine is the main component. The objective was to evaluate the effects of guarana and caffeine on MSCs. After the initial characterization, MSCs were treated with *Paullinia cupana* (10, 100, and 1000 μg/mL) or caffeine (0.4, 4, and 40 μg/mL) for 24 h. MSCs treatment with 1000 μg/mL guarana increased cell polarity, viability, cell migration to chemoattractant, antioxidant potential, and liberation of extracellular vesicles (EVs), while it reduced the levels of autophagy. MSCs treated with 100 and 1000 μg/mL guarana or 40 μg/mL caffeine showed a decrease of cell proliferation. No treatment affected the cellular area and cell cycle of MSCs. The study shows *in vitro* evidence that guarana could be a promising alternative for activating MSCs to promote better cellular products for future clinical therapies.

## Introduction

Mesenchymal stromal cells (MSCs) have been investigated as a potential alternative for cell therapy due to their characteristics of self-renewal, differentiation, and, mainly, their paracrine immunomodulatory and immunosuppressive effect. Currently, different approaches to enhance the recruitment of MSCs and their anti-inflammatory capabilities attract significant interest. Thus, the activation of MSCs with cytokines, growth factors, hypoxia, pharmacological drugs, biomaterials, among other different culture conditions, mechanisms also known as activation or “priming”, are extensively investigated to generate cellular products with greater potential for the different clinical applications (1).

Guarana (*Paullinia cupana*) is a native plant from the central Amazon basin, found in South American countries, known for its stimulating and medicinal properties. Roasted seed extracts have been used as medicinal beverages for centuries by indigenous communities (2). Seeds are the commercially useful part of the plant due to their high content of the purine alkaloid caffeine (1,3,7-trimethylxanthine), to which its stimulant property is attributed. Despite the variation in caffeine content of the seeds (from 2.5 to 6%), the levels are still high compared to any other species, including coffee (*Coffea arabica*), tea (*Camellia sinensis*), and yerba mate (*Ilex paraguariensis*) (2). Guarana seed extract is composed mainly of caffeine (34.19 mg/g of extract), theobromine (0.14 mg/g), catechin (3.76 mg/g), and epicatechin (4.05 mg/g) (3).

Previous investigations describe antidepressant, antimicrobial, anti-platelet aggregation, antioxidant, and cardioprotective effects of guarana (4,5). The antioxidant effects of guarana have been evaluated in fibroblasts from NIH-3T3 cell line, in which guarana decreased cell mortality, lipid peroxidation, DNA damage, and cell oxidative stress and increased superoxide dismutase (SOD). These levels demonstrate the effects on nitric oxide (NO) metabolism in situations with high cellular NO levels (6). Besides that, fibroblasts from human cell line HFF-1 responded to guarana treatment by increasing interleukin (IL)-10 levels, while not producing inflammatory cytokines (7). Additionally, guarana possibly protects skin cell lines and fibroblasts from cytotoxic effects (8). In neuronal SH-SY5Y human cells, an increase in the non-enzymatic antioxidant potential was demonstrated (9). Furthermore, *in vivo* studies demonstrated anti-aging effects of guarana in treatment models of Alzheimer's and Huntington diseases, mediated by antioxidant activity and DAF-16, HSF-1, and SKN-1 pathways (10,11), and neuroprotective effects in hyperlipidemia rat disorder (12). Finally, *in vitro* studies with MSCs and the guarana components theobromine and caffeine demonstrate an increase in osteogenic potential (13) and a beneficial effect on cultured primary adipose-derived stem cells and bone marrow stromal cell line (M2-10B4), increasing differentiation into osteoblasts mediated by the activation of RUNX2 (14) at low doses; however, this effect is significantly mitigated in high doses.

Since a previous investigation has described several biological properties of guarana in cell lines, in this study we analyzed the effects of guarana and caffeine on MSCs to assess the possibility of using this extract as a priming approach for cell therapy.

## Material and Methods

### Ethical statement

This study was approved by the Research Ethics Committee of Hospital de Clínicas de Porto Alegre (HCPA 2019-0587, CAAE 33841720.5.0000.5327). All donors of chorionic membrane provided written informed consent.

### Isolation, characterization, and culture of MSCs

MSCs were isolated from full-term human chorionic membrane provided by healthy donors at the Hospital de Clínicas de Porto Alegre, Brazil. The tissues were processed according to Araújo et al. (15). Segments of placenta containing neonatal and maternal tissues were separated aseptically and immediately transported to the laboratory in sterile saline for processing (time from collection to processing was <1 h). Forceps and scalpel were used to separate neonatal and maternal placental tissues. The chorionic membrane was washed thoroughly with saline solution, fragmented into small pieces, and 1.5-2.5 g was used to continue the protocol. The tissue pieces were digested with type I collagenase (1 mg/mL, Gibco, USA) for 2 h at 37°C with agitation every 10 min. After adding fetal bovine serum (10% FBS; Gibco) for enzymatic neutralization, filtration was carried out using a 100-µm filter (BD Falcon, Norway), followed by centrifugation at 500 *g* for 6 min at 20°C. Obtained cells were cultured in 6-well plates in Dulbecco's Modified Eagle Medium (DMEM; Gibco), 20%FBS/1% penicillin-streptomycin solution (PS; Gibco)/2 mM L-glutamine (Sigma-Aldrich, USA) at 37°C, in 5% CO_2_ humid atmosphere. Culture medium was changed twice a week, until 80-90% confluence. At this point, cells were detached using 0.25% trypsin-EDTA solution and expanded for the experiments.

Cells were characterized by immunophenotype analysis and by *in vitro* differentiation in mesodermal lines. Immunophenotype analysis was performed by flow cytometry (FACSCanto II cytometer, BD Biosciences (USA); FlowJo software, FlowJo LLC, USA). CD73, CD90, CD105, CD44, CD45, CD34, CD11b, CD19, and HLA-DR were assessed (BD Stemflow hMSC Analysis Kit, BD Biosciences). Expression of CD29 (BD Pharmigen, BD Biosciences), CD14 (BD Pharmigen, BD Biosciences), CD3 (Exbio), HLA-G (Exbio), and CD56 (BD Pharmigen, BD Biosciences) were also performed. Related isotype antibodies were used as a control. Differentiation induction was carried out in osteocytes (10-14 days), adipocytes (28 days), and chondrocytes (28 days) using STEMPRO differentiation kit (Gibco). Medium was changed twice a week. Alizarin Red staining was used to confirm calcium mineralization in osteogenic induction, Oil Red was used to confirm adipogenic differentiation, and Alcian Blue was used to confirm chondrogenic potential.

### Treatments and experimental design *in vitro*

MSCs (P3-P7) from three different donors were individually cultivated with different treatment medium, considering seven experimental groups: 10, 100, and 1000 μg/mL guarana in DMEM 10% FBS/1% PS (G10, G100, and G1000, respectively); 0.4, 4, and 40 μg/mL caffeine in DMEM 10% FBS/1% PS (C0.4, C4, and C40, respectively). MSCs cultured with DMEM 10% FBS/1% PS were used as the control group. Cells were treated for 24 h before assessments (3).

Guarana stock solution (10 mg/mL) was prepared using powdered guarana extract [Sanitas, Lifar (Lot 1884A19), Brazil] diluted in DMEM, supplemented with 10% FBS in 1% PS, incubated at 37°C for 15 min, and subsequently filtered with a syringe filter (0.22 μm). The guarana treatment solutions were diluted to concentrations of 10, 100, and 1000 µg/mL according to Bittencourt et al. (3). As caffeine is the main component of guarana extract, the caffeine concentrations corresponding to each guarana solution were tested. For the stock solution (1 mg/mL), anhydrous caffeine (99%; Sigma-Aldrich) was diluted in DMEM 10% FBS/1% PS and filtered with a syringe filter (0.22 μm). The caffeine treatment solutions were diluted at concentrations of 0.4, 4, and 40 µg/mL. The culture medium treatment solutions were maintained at 4°C.

### Area, cell polarity, and nuclear morphology

MSCs were plated (4×10^4^ cells/well) onto coverslips in a 12-well plate. After the treatment period, samples were washed with phosphate-buffered saline (PBS; Laborclin, Brazil) and fixed with 4% paraformaldehyde solution + 4% saccharose for 15 min at room temperature (RT). After washing with PBS, the membranes were permeabilized with 0.3% Tween (Sigma-Aldrich) in PBS for 10 min, washed, and blocked with FBS (1:10) for 1 h at RT. Subsequently, cells were labeled with rhodamine-phalloidin (Invitrogen, Thermo Fisher, USA ) for 1 h at 4°C. Nuclei were stained with DAPI (4',6-diamidino-2-phenylindole; Sigma-Aldrich) for fluorescence microscopy analysis.

Cell area was measured using the ImageJ software (http://imagej.nih.gov/ij). To assess cell polarity, the polarity index was estimated as the length of the major migration axis (parallel to the membrane protrusion) divided by the length of the perpendicular axis that intersects the center of the cell nucleus (16,17). Approximately 100-150 cells were analyzed in each condition.

Nuclear morphometric analysis (NMA) was performed as described by Filippi-Chiela et al. (17). Images were taken from 300-400 nuclei, obtained from random fields. Images were analyzed using Image Pro-Plus 6.0 (Media Cybernetics, USA) for the acquisition of the nuclear area, and the parameters of nuclear irregularity (roundness, aspect, radius ratio, and area/box) were grouped based on the nuclear irregularity index (NII). NMA was used to quantify senescence, mitosis, apoptosis, and mitotic catastrophe.

### Cell viability (MTT)

MSCs (1×10^4^/well) were seeded onto a 96-well plate and exposed to the treatment conditions. After 24 h, the treatment medium was changed to a standard medium containing 3-(4,5-dimethylthiazol-2-yl)-2,5-diphenyltetrazolium bromide (MTT, Sigma-Aldrich) at the final concentration of 0.5 mg/mL. After 4 h, the medium was removed and DMSO was added. The 570 nm and 690 nm absorbances were used in the spectrophotometer (Spectramax, Hexis, USA) (16).

### Mitochondrial membrane potential and quantification (MitoTracker Red FM)

Onto 24-well plates, 4×10^4^ MSCs were seeded. After 24 h of treatment, cells were washed with PBS, detached with a trypsin/EDTA solution, and a labeling mix containing DMEM 10% FBS and MitoTracker^®^ Red FM was added. After incubating for 20 min/37°C, PBS was added, and the samples were analyzed on Attune flow cytometer (ThermoFisher Scientific, Singapore), according to protocol adapted from Keil et al. (18).

### Mitochondrial membrane potential (JC-1)

Onto 24-well plates, 4×10^4^ MSCs were seeded. After 24 h of treatment, cells were washed with PBS, detached with a trypsin/EDTA solution, and a labeling mix containing DMEM 10% FBS and tetraethyl-benzimidazolyl-carbocyanine iodide (JC-1 - ab113850) was added. After incubating for 20 min at 37°C, PBS was added, and the samples were analyzed on Attune flow cytometer (ThermoFisher Scientific), according to protocol adapted from Keil et al. (18).

### Antioxidant potential

To evaluate antioxidant potential, total SH (reduced thiol) groups of proteins and other thiol compounds were measured. MSCs (30×10^4^/well) were plated onto a 6-well plate. After the treatments, cells were detached, resuspended in PBS, and centrifuged (3000 *g*/10 min at 4°C). Then, the supernatant was used to determine total SH groups as described by Silveira et al. (19). Briefly, 30 to 80 µg aliquot was diluted in phosphate-buffered saline and reacted with 10 mM 5,5'-dithiobis-(2-nitrobenzoic acid). After 60 min at RT, the absorbance was measured using a spectrophotometer (412 nm). Results are reported as mmol-SH groups/mg protein.

### Cell proliferation (population doubling)

MSCs were plated at a density of 4×10^4^ cells/well onto a 24-well plate and treated. On days three and six, cells were detached, counted using a Neubauer's chamber, and re-seeded at the same initial concentration (4×10^4^ cells/well). To obtain the population doubling (PD) values, the initial and final values of cells in each time interval were entered into the equation: PD = (log N(t) - log N(t0)) / log 2, where N(t) was the number of cells per well at the time of trypsinization after three and six days and N(t0) was the number of cells initially plated, which was adapted from Silveira et al. (19).

### Autophagy and cell cycle characteristics

To analyze autophagy, 4×10^4^ cells/well were seeded onto 24-well plates. After the treatment period, cells were washed with PBS, trypsinized, and resuspended in 400 μL of DMEM 10% FBS + 0.6 μL acridine orange (Sigma-Aldrich). Then, the plates were incubated for 15 min at RT, protected from light, and analyzed on the Attune flow cytometer (ThermoFisher Scientific), according to protocol adapted from Silva et al. (20).

For cell cycle analysis, after guarana or caffeine treatments, cells were washed with PBS, trypsinized, and centrifuged at 300 *g* for 10 min at RT. Then, cells were resuspended in 0.5 μg/mL propidium iodide (Sigma-Aldrich), 0.1% Triton x-100, and 0.1% sodium citrate solution, agitated, and incubated for 15 min at 4°C in the dark. Finally, the nuclei were analyzed in the flow cytometer, according to protocol adapted from Silva et al. (20).

### Wound healing and transwell migration assay

Onto 24-well plates, 5×10^5^ cells were seeded and after 24 h of treatment, a 200 µL tip was used to remove a defined number of cells from the cell monolayer, creating a wound. To evaluate the random migratory behavior of MSCs, a phase contrast microscope (Olympus, IX71/Olympus DP71 camera, Japan) was used to monitor the cells at 0, 12, and 24 h after the wound. Five photos were taken in random fields of each well and the gap area was measured with the ImageJ software (http://imagej.nih.gov/ij) (5 measurements in each photo).

To analyze the migratory behavior of MSCs towards a chemoattractant, a transwell assay was performed, according to protocol adapted from Schneider et al. (16). A membrane transwell culture (8 µm; Corning, Sigma-Aldrich, USA) was used, and DMEM supplemented with 10% FBS was previously plated on the upper chamber for migratory stimulation. MSCs were previously cultured with guarana or caffeine (1000 and 40 µg/mL, respectively) for 24 h, and then 1×10^5^ cells/insert were transferred to the upper chamber. The chemoattractant SDF-1 (stromal cell-derived factor 1; Sigma-Aldrich; 300 ng/mL) was added to the lower chamber, and inserts were incubated for 16 h. The medium was removed and the inserts were washed with PBS, the cells were fixed with 4% paraformaldehyde at RT for 15 min, and washed again with PBS. Then, MSCs were stained with HE (hematoxylin-eosin) and cells from the upper chamber were removed using a cotton-tipped swab. Migrated cells were counted using an inverted microscope (Olympus) and the migratory behavior was calculated as the ratio of the number of cells moving towards SDF-1 (control group) to the number of cells moving after guarana and caffeine treatments.

### Isolation of extracellular vesicles

After treating the cells for 24 h with 1000 μg/mL guarana or 40 μg/mL caffeine, the supernatant was collected, and a differential centrifugation was used to isolate the extracellular vesicles (EVs). First, cells were removed by centrifugation at 300 *g* for 30 min at 4°C. Subsequently, the supernatant was filtered (0.2 µm) to remove large particles. The filtered supernatants were ultracentrifuged (100,000 *g* for 2 h at 4°C; Hitachi WX ultracentrifuge, angle rotor P40ST -1551, Japan). EVs were collected in 1000 µL of filtered PBS according to Franquesa et al. (21).

Analysis of the absolute size distribution of EVs was performed using NanoSight (NTA 3.2 Dev Build 3.2.16, UK). With NTA, particles were analyzed based on Brownian motion and diffusion coefficient using a red laser. EVs were diluted (1-50) in 1 mL of filtered PBS, and the NTA measurement conditions were 24.50±0.5°C and measurement time 60 s. Two recordings were made for each sample.

### Statistical analysis

Statistical analysis was performed with GraphPad INSTAT (GraphPad Software, USA), version 8. Data are reported as means±SD unless otherwise indicated. Differences between groups were evaluated by either one-way analysis of variance (ANOVA) followed by Tukey's honest significant difference test for normally distributed variables or Kruskal-Wallis test with Dunn's multiple comparison test for non-normal samples. P<0.05 was considered statistically significant. All data were collected from three independent experiments.

## Results

### Characterization of MSCs from chorionic membrane

Chorion-derived MSCs from human placenta with stable fibroblast-like phenotype were isolated by adherence separation and characterized by flow cytometry and differentiation assays. Cells expressed MSCs markers CD73 (99.2±0.6), CD90 (96.6±0.4), CD105 (92.8±7.0), CD29 (97.1±0.5), and CD44 (96.0±2.3) and lacked expression of CD45-CD34-CD19-CD11b-HLA-DR (0.2±0.1), CD14 (0.4±0.3), CD3 (0.3±0.3), and HLA-G (1.6±0.6). MSCs demonstrated capacity to differentiate into osteocytes, adipocytes, and chondrocytes after incubation in appropriate differentiation media from 14 to 28 days. These results were consistent with human MSCs.

### Morphometric analysis: morphology, size, polarity, and nuclear regularity

**Figure 1:**
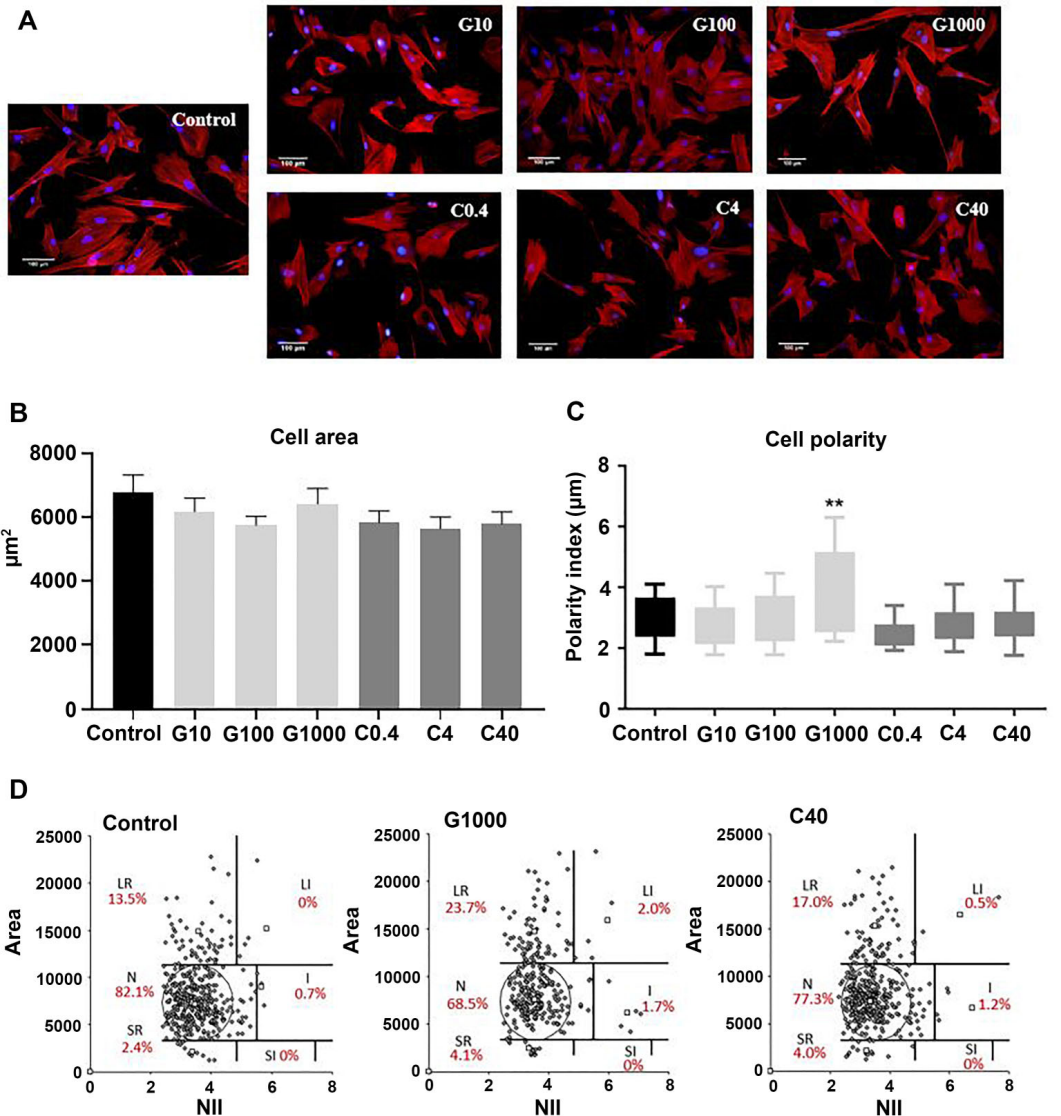
Morphometry analysis of mesenchymal stromal cells (MSCs). **A**, Human MSCs morphology after 24 h treatment with guarana (*P. cupana*) or caffeine. MSC stained with Rhodamine-phalloidin and DAPI (scale bar 100 μm). **B**, Cell area, with no significant differences between groups. **C**, Cell polarity index. G1000 cells showed the most elongated morphology, presenting the highest polarity index (4.065±1.318) (**P<0.01 compared to control and G100; **P<0.001 compared to G10 and all caffeine groups). **D**, Nuclear morphometric analysis by the nuclear irregularity index (NII). Numbers indicate the percentage of nuclei in each category (N: normal; S: mitotic; SR: apoptotic; SI: small and irregular, LR: senescent; LI: large and irregular; I: irregular). Data are reported as means±SE (n=3 in 3 independent experiments). Statistical analysis was performed using one-way ANOVA and Tukey's *post hoc* multiple comparison test. G: guarana 10, 100, and 1000 μg/mL; C: caffeine 0.4, 4, and 40 μg/mL.

MSCs maintained their characteristic fibroblastic morphology (Figure 1A) after guarana and caffeine treatments in different concentrations. Regarding cell area, no significant differences were observed compared to the control group (P>0.05) (Figure 1B).

When polarity was analyzed (Figure 1C), cells treated with 1000 μg/mL guarana showed the most elongated morphology, presenting a higher polarity index (4.065±1.318) (P<0.01 compared to control and G100; P<0.001 compared to G10 and all caffeine groups). NMA analysis showed no biologically relevant modifications in the cell nucleus among different treatment groups (Figure 1D).

### Cell viability - MTT

**Figure 2:**
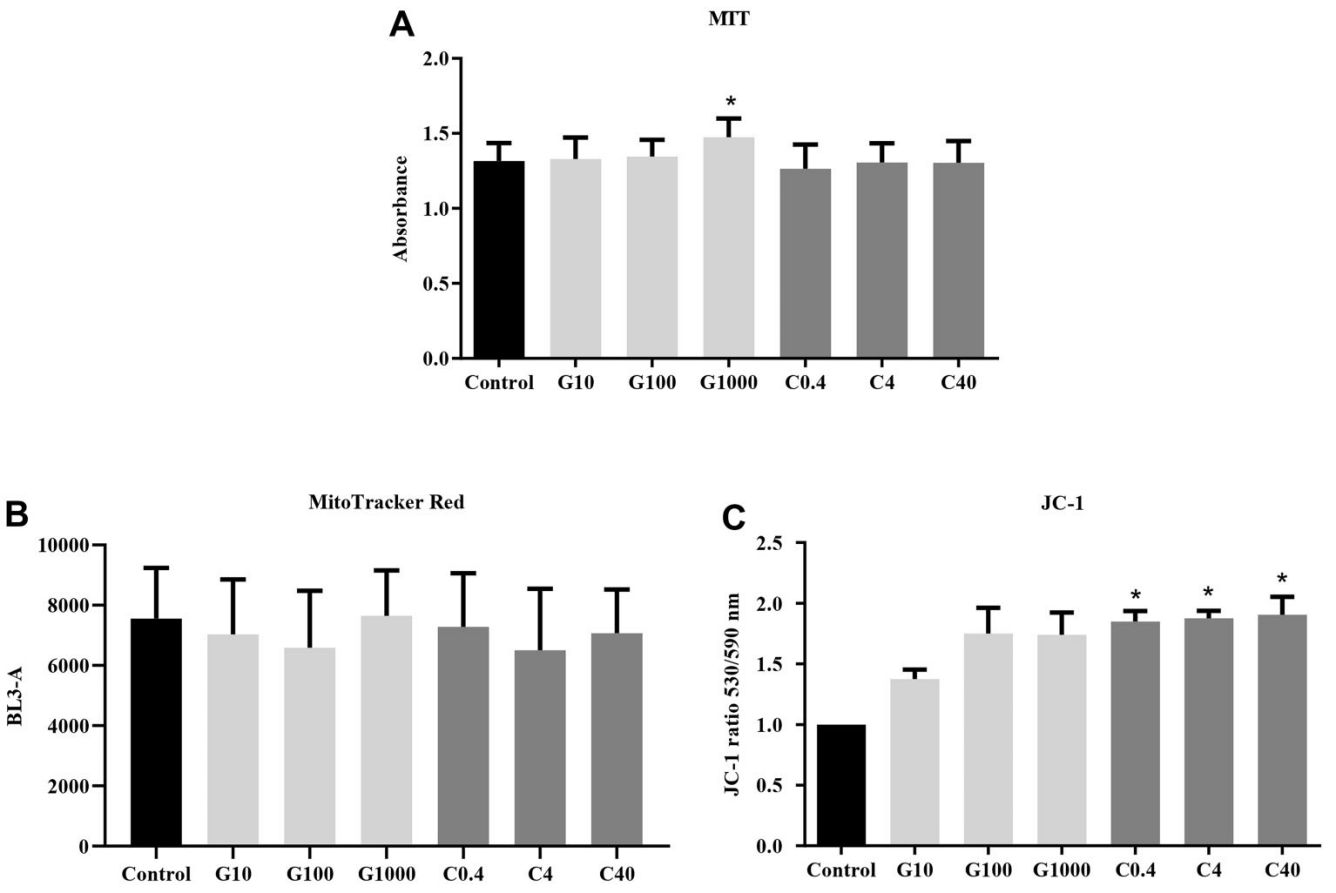
Cell viability analysis. **A**, MTT assay of mesenchymal stromal cells (MSCs) after guarana or caffeine treatment. *P=0.04 compared to groups C4, C40, and C0.4. **B**, MitoTracker Red membrane potential and mitochondria quantification presented no differences between groups. **C**, JC-1 mitochondrial membrane potential of MSCs. Data are reported as means±SE. *P=0.03 compared to control group. Analysis was performed using one-way ANOVA and Tukey's *post hoc* multi-comparison test for **A** and **B** and Kruskal-Wallis test with Dunn's multiple comparison test for **C** (n=3 in 3 independent experiments). G: guarana 10, 100, and 1000 μg/mL; C: caffeine 0.4, 4, and 40 μg/mL.

Viability of treated cells with guarana or caffeine was measured and Figure 2A shows the results. MTT assay demonstrated that the treatment with guarana at 1000 μg/mL provided significantly higher viability (1.475±0.125) compared to groups C4 and C40 (P<0.01), as well as control and C0.4 (P=0.04).

### Mitochondrial membrane potential - Mitotracker Red FM and JC-1

After demonstrating that *P. cupana* induced greater cell viability (measured by mitochondrial conversion of MTT), the mitochondrial membrane potential was evaluated by flow cytometry. Using the MitoTracker Red assay, no statistical difference in the number of mitochondria and membrane potential were observed among groups (Figure 2B). JC-1 assay resulted in green fluorescence when cells presented low mitochondrial membrane potential and red fluorescence when mitochondria presented high membrane potential. The red/green fluorescence ratio was used to determine the mitochondrial function of cells after treatments (Figure 2C). Caffeine (C0.4, C4, and C40) groups promoted an increase in mitochondrial membrane potential being significantly more polarized (P=0.03) than the control.

### Antioxidant potential

**Figure 3:**
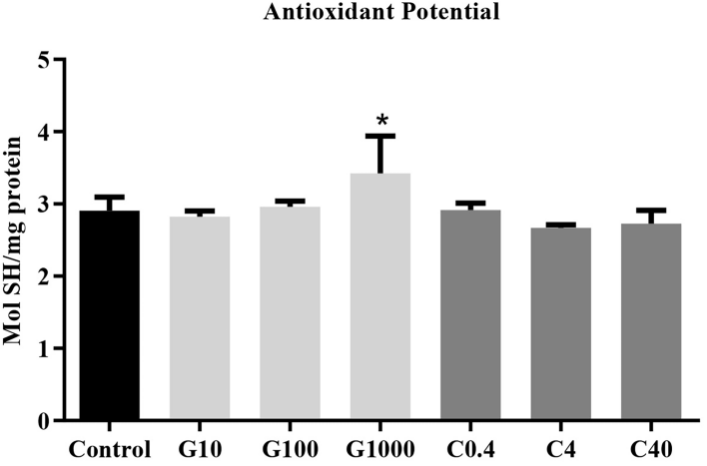
Antioxidant potential. Quantity of reduced thiols (SH) per mg of protein after 24 h of treatment with guarana or caffeine (n=3 in 3 independent experiments) of mesenchymal stromal cells. G: guarana 10, 100, and 1000 μg/mL; C: caffeine 0.4, 4, and 40 μg/mL. Data are reported as means±SE. *P=0.02 compared to caffeine groups (ANOVA).

The process of oxidative stress occurs naturally in cells and causes cell death. Our results demonstrated that treatment with 1000 μg/mL guarana increased the number of SH (P=0.02) (Figure 3). This may provide cells with protective effects against oxidative changes of proteins.

### Cell proliferation - PD

**Figure 4:**
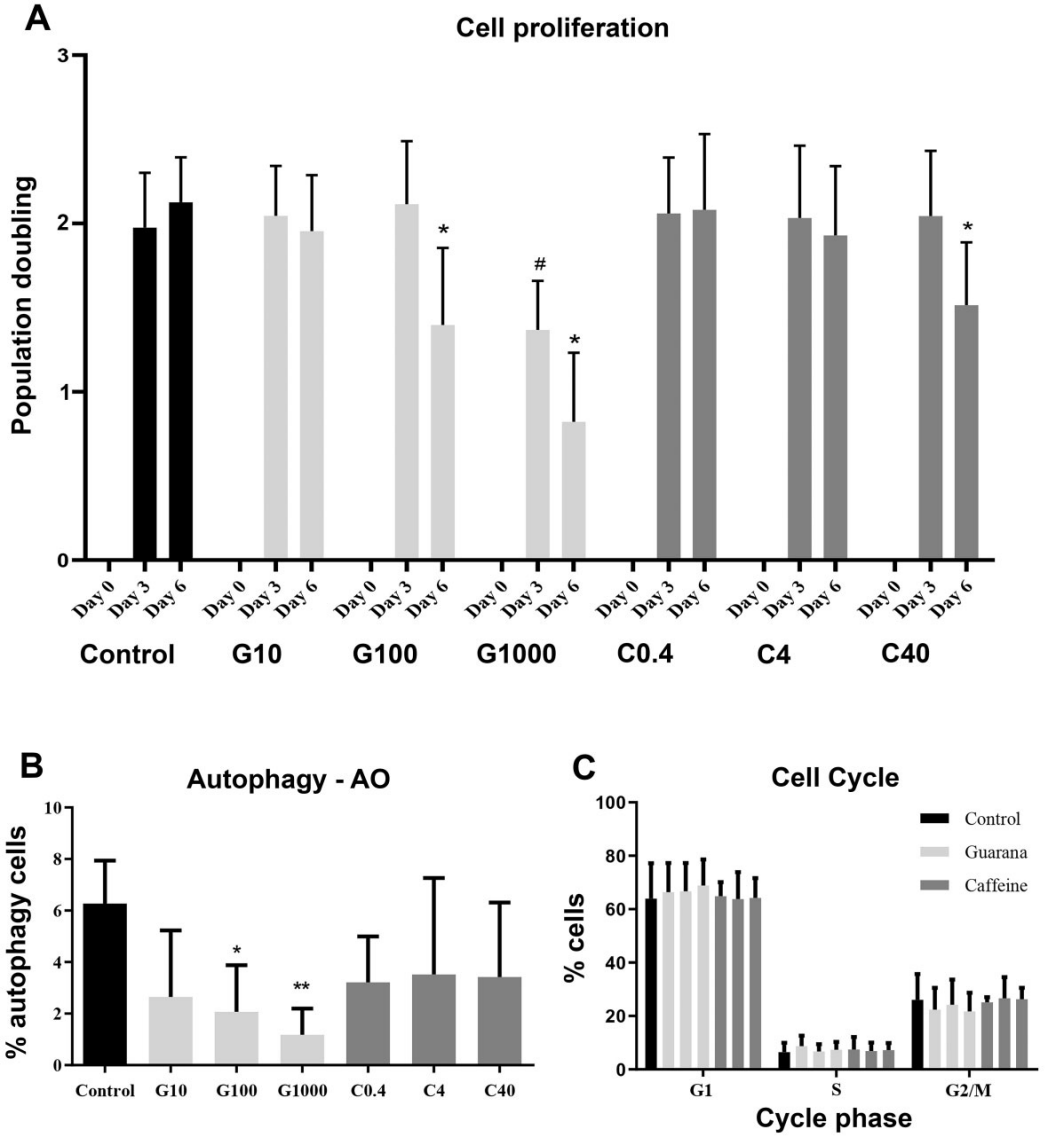
Growth kinetics of mesenchymal stromal cells (MSC). **A**, Number of population doublings (PD) after treatments with guarana or caffeine. On day three, ^#^P=0.0426 compared to the control group; on day six, the groups G1000, G100 (*P<0.0001), and C40 (*P=0.0120) showed lower proliferation compared to control group. **B**, Percentage of autophagic cells stained with acridine orange after 24 h of guarana or caffeine treatments. G1000 (**P=0.0041) and G100 (*P=0.0143) were different from the control group. **C**, MSC cell cycle after 24 h of guarana or caffeine treatment. No differences were observed among groups (P>0.05). Data are reported as means±SE. Two-way and one-way ANOVA followed by Tukey's *post hoc* multiple comparison test were performed (n=3 in 3 independent experiments). G: guarana 10, 100, and 1000 μg/mL; C: caffeine 0.4, 4, and 40 μg/mL.

The proliferation assay demonstrated that guarana at 100 and 1000 μg/mL concentrations and caffeine at 40 μg/mL promoted lower PD values in relation to the control group (Figure 4A). On day three, there was a lower PD in G1000 compared to control group (P=0.043); on day six, the groups G1000, G100 (P<0.0001), and C40 (P=0.0120) showed lower proliferation compared to the control group.

### Autophagy and cell cycle

MSCs treated with 1000 μg/mL guarana presented lower levels of autophagy (1.320±0.470%) compared to control (6.530±0.718%) (P=0.004) (Figure 4B). Moreover, the MSC cell cycle was analyzed using flow cytometry and showed no difference among all groups. Treatments with guarana and caffeine did not change the percentage of cells in the G0/G1, S, or G2-M phases (P<0.05) compared to the control group (Figure 4C).

### Cell migration

**Figure 5:**
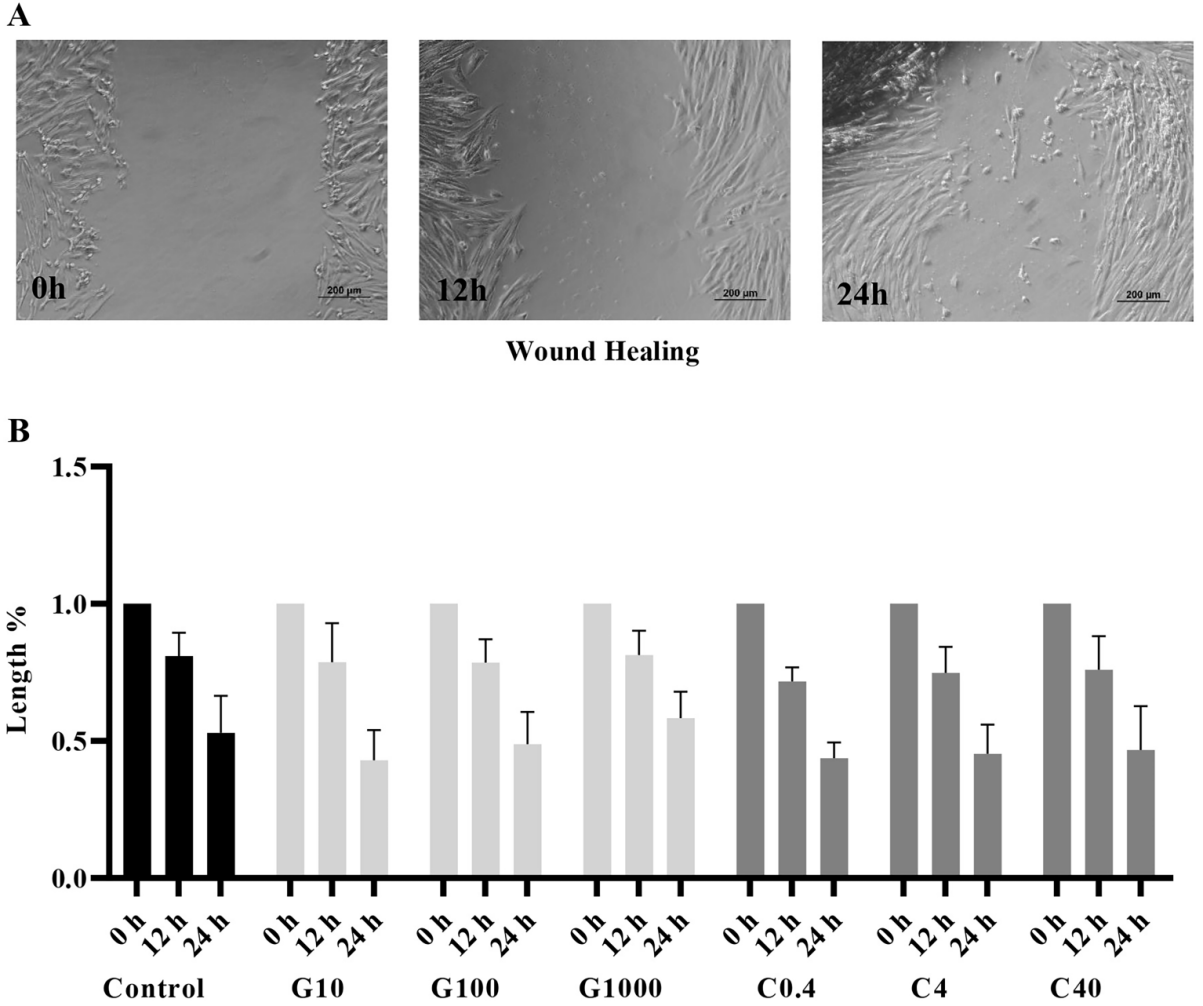
Wound healing analysis. **A**, Representative images from the control group of untreated mesenchymal stromal cells. The reduction of the wound over time can be observed (scale bar 200 μm). **B**, No differences were observed in the length of the wound when different treatments and the control group were compared (P>0.05). Two-way ANOVA and Tukey's *post hoc* multiple comparison test were performed (n=3 in 3 independent experiments). G: guarana 10, 100, and 1000 μg/mL; C: caffeine 0.4, 4, and 40 μg/mL.

**Figure 6:**
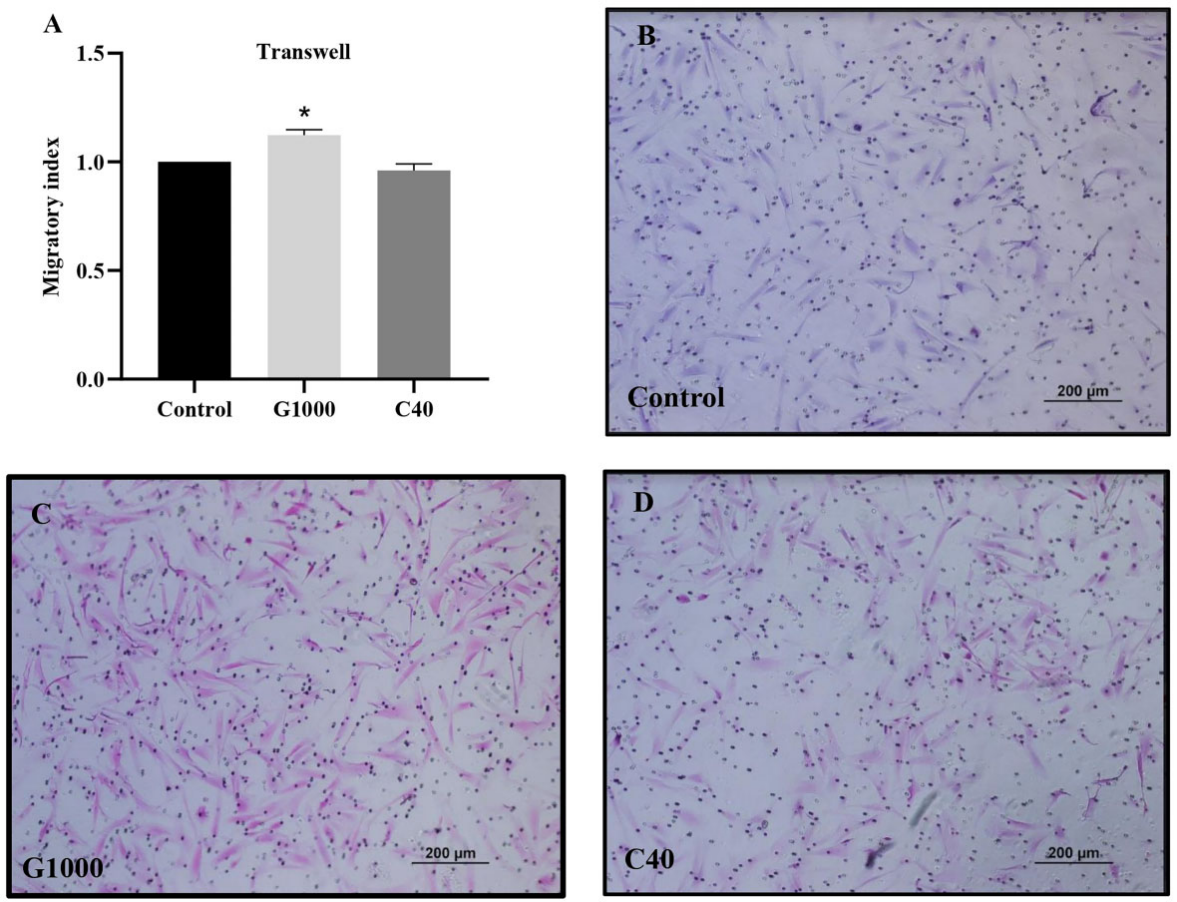
Transwell assay. **A**, Migratory index of mesenchymal stromal cells (MSCs) calculation demonstrated that G1000 presented a higher migration toward SDF-1 compared to control group (*P=0.0191). **B**-**D**, Representative images of migrated MSCs stained with hematoxylin-eosin. Scale bar 200 μm. Analysis was performed using Kruskal-Wallis test with Dunn's multiple comparison test (n=3 in 3 independent experiments). G1000: guarana 1000 μg/mL; C40: caffeine 40 μg/mL.

The wound healing assay is used to assess random cell migration, and no differences were observed among groups (Figure 5). However, in the transwell assay, when a specific chemoattractant for MSCs was used, the G1000 group had a significantly higher migratory rate compared to control (P=0.019) (Figure 6).

### Extracellular vesicle isolation

**Figure 7:**
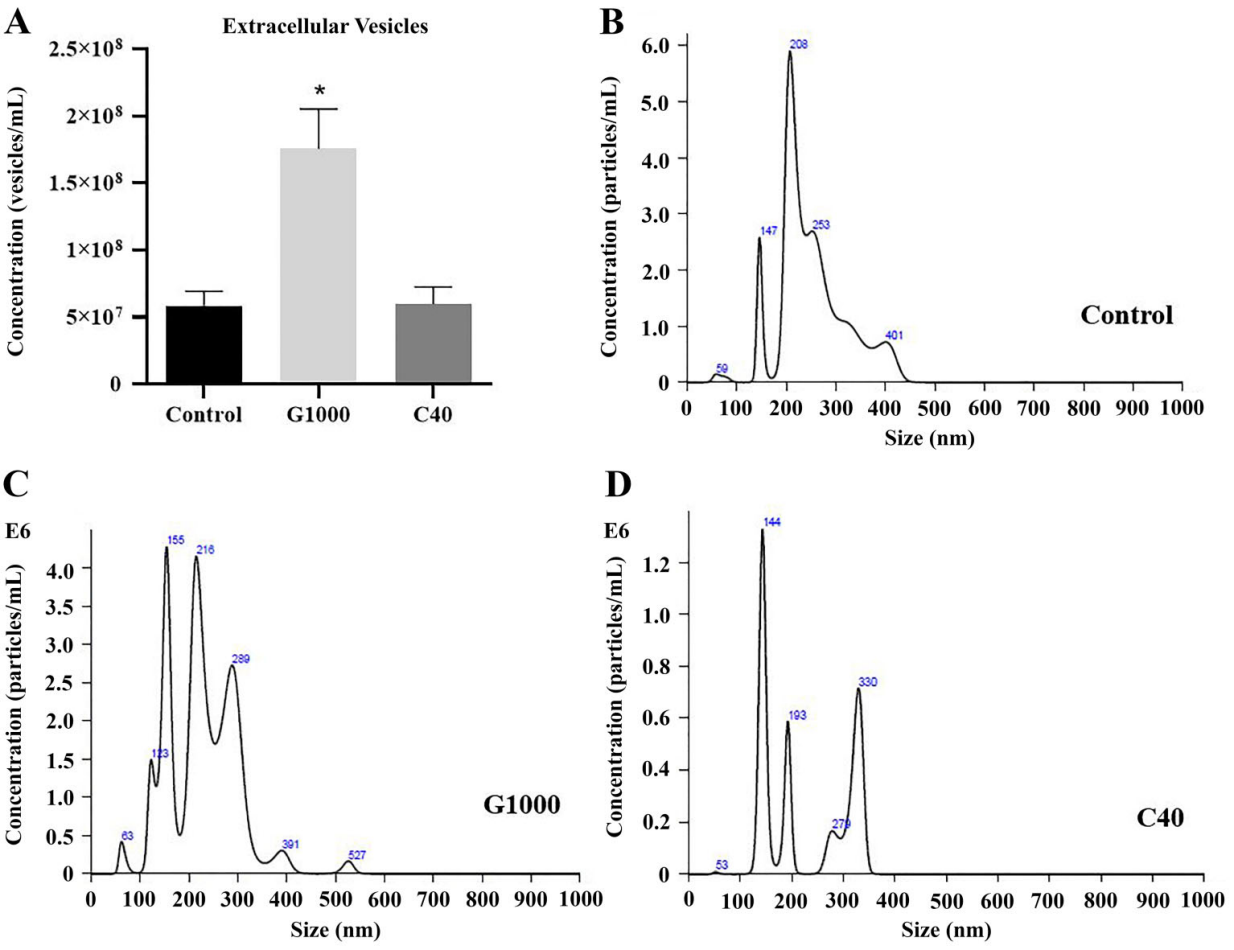
Extracellular vesicles (EVs) secreted by mesenchymal stromal cells (MSCs) under guarana or caffeine treatments. **A**, EVs quantification - G1000 increased the release of EVs compared to the control and C40 groups (*P<0.05). EVs Analysis was performed using Kruskal-Wallis test with Dunn's multiple comparison test. **B**-**D**, Analysis of the absolute size distribution of EVs was performed using NanoSight. Conditioned medium was collected from wells where 4×10^4^ cells/well were seeded (n=3 in 3 independent experiments). G1000: guarana 1000 μg/mL; C40: caffeine 40 μg/mL.

After analyzing the isolated extracellular vesicles, including exosomes (30-100 nm), microvesicles (100-1000 nm), and apoptotic bodies (50-5000 nm), treatment with 1000 µg/mL guarana increased the release of EVs compared to the control group and C40 groups (P<0.05) (Figure 7).

## Discussion

The therapeutic potential of MSCs is related mainly to lineage-specific differentiation, immune system modulation, secretion of bioactive factors, and their antioxidant properties. These properties can be tightly regulated by biological, biochemical, and biophysical factors that can enhance MSC therapeutic potential (22). Guarana presents high content of polyphenols and caffeine and has demonstrated strong antioxidant and anti-inflammatory properties (6,7,9-11,23). In this context, we evaluated MSCs viability, cell and nuclear morphometry, cell polarity, cell cycle, autophagy, mitochondrial membrane potential, as well as antioxidant properties in the presence of guarana extract, aiming to apply this natural product as a MSCs priming agent. Moreover, we included caffeine-independent analysis to evaluate if guarana extract effects are related to the presence of caffeine.

In the morphometric analysis, our data demonstrated that cells treated with guarana and caffeine had no differences compared to the control group. Regarding cell polarity, using a polarity distribution index, we demonstrated that 24 h of guarana treatment promoted a more elongated cell shape. Cell polarity results from the internal organization of the cell and is a key step for the induction of cell motility. Cell migration depends on the activation of several signaling pathways that result in the break of cell asymmetry, where a more elongated morphology is usually associated with a better mesenchymal cell migratory performance (9). Schneider et al. (16) demonstrated, using time-lapse microscopy, which random migration correlates with polarity index data, since elongated cells presented a significant increase in cell speed and spatial trajectory compared to round-shaped MSCs. Moreover, Geissler et al. (24) observed that chronological and *in vitro* aging leads to round-shaped MSCs and diminished migration potential, as well as a decrease in the expression of genes associated with cytoskeletal organization.

Considering that the homing process is a key step for MSCs to participate in tissue repair and immunomodulation, the modification of the polarity index promoted by guarana could affect cell migration, contributing to cell therapy. To test if guarana could promote better migratory behavior, we conducted wound-healing and transwell assays. We observed no differences among groups in the wound-healing assay. However, during the wound-healing assay, the cells migrate into the gap and proliferate, possibly interfering with the measurement of migration (25). Notably, the G1000 group presented the highest polarity index, but the lowest proliferation, which possibly affects gap closure. Homing of mesenchymal stromal cells is mediated by the interaction of chemokines with receptors in the plasma membrane that signal their migration to the injured sites. To address this important MSC function, we performed a transwell assay with SDF-1 and observed enhanced responsiveness to SDF-1 in MSCs cultivated or primed with G1000. Our data corroborate the findings of Lim et al. (26), who demonstrated that different priming agents affect MSC migration.

Mitochondria are the main energy-producing organelles and play a key role in the regulation of cellular bioactivity. Their transmembrane potential is eventually lost after significant cellular damage (27). Lu et al. (28) has observed improvements in mitochondrial membrane potential in activated MSCs and this was associated with better therapeutic effects. We demonstrated that guarana, as well as caffeine, preserved mitochondrial membrane potential and that caffeine promoted a significant hyperpolarization. Moreover, guarana (1000 μg/mL) promoted an increase in cell viability by MTT assay, which is a mitochondrial dehydrogenase in the living cells. Guarana bioactive compounds can also modulate the expression of mitochondrial biogenesis-related genes (29). This is especially interesting because it is known that MSC also act through the transfer of healthy organelles (including mitochondria) to host cells (30).

Keil et al. (18) found that mitochondria are irregularly distributed, resulting in regions with higher or lower mitochondrial content, when studying mitochondrial membrane potential markers in astrocytes. Furthermore, the authors confirmed that JC-1 would be a more suitable marker for cellular heterogeneity, as it can differentiate functional and depolarized mitochondria by the green/red ratio. Moreover, Buravkov et al. (31) observed that when using JC-1, the changes occur mainly in the red band. On the other hand, the MitoTracker Red FM is insensitive to repeated laser irradiation, where the intensity of its fluorescence in the mitochondria in the presence of CCCP (uncoupler of mitochondrial oxidative phosphorylation) decreased by 2,125-fold, while it remained practically unchanged in cytoplasm. Our results corroborated the above data, since MitoTracker Red FM findings were not significantly different, while a significant difference was found in the JC-1 analysis, probably because it is the most suitable marker for the heterogeneous characteristics of MSCs of primary culture.

Changes in cell viability could result in a diminished number of cells and a decreased effectiveness of cell therapy. We demonstrated that guarana and caffeine did not impair the viability of human-derived MSCs in most of the studied concentrations. Moreover, guarana at the highest concentration (G1000) increased cell viability. Nuclear morphometry can indicate several cell outcomes such as early apoptosis and senescence, which may compromise MSC quality for therapeutic purposes. We observed no significant difference in nuclear morphometry or cell cycles, indicating that the treatments studied were not toxic for MSCs. Nonetheless, Zeidán-Chuliá et al. (9) reported that 12.5-50 mg/mL guarana and its combination with caffeine promoted apoptosis, including membrane blebbing, cell shrinkage, and cleaved caspase-3 positivity in human neuronal SH-SY5Y cells. Similar to our data, guarana (10, 100, and 1000 μg/mL) decreased induced toxicity in human neuronal‐like cells (3) and showed protective effects in NIH-3T3 (6) and HFF-1 fibroblasts (7). In addition, Aldhahrani (32) demonstrated that oral administration of guarana seed extract impacted apoptosis-related gene expression induced by methotrexate, a medication known for its high organ toxicity. Moreover, the impact on gene expression indicates an anti-apoptotic effect exerted by guarana.

Autophagy is crucial for maintenance of bioenergetics and cell viability *in vitro* and plays an essential role in survival of the whole organism. *In vitro*, human MSCs showed a high level of constitutive autophagy, which can be up- or downregulated in stress or differentiation (33). We observed that 1000 µg/mL guarana significantly reduced autophagy levels. Remarkably, De Witte et al. (34) recently demonstrated that priming MSCs with interferon (IFN) promotes the methylation of HS1BP3, a gene implicated in the regulation of MSC autophagy.

We also showed that higher concentrations of guarana extract provided higher levels of SH, demonstrating that the antioxidant effects of *P. cupana* observed in cell lines are also present on MSCs. This effect may be associated with the presence of caffeine, theobromine, tannins, catechins, and epicatechins (35). However, in our study, the treatment with proportional caffeine concentration presented similar effects to the control group, confirming that other guarana compounds are needed to promote higher levels of antioxidant potential. This cellular antioxidant effect can reduce apoptosis and other damages induced by oxidative stress (36).

We observed a significant increase in the release of EVs from G1000 group. MSC-EVs can carry a variety of molecules with therapeutic functions. Many reports have highlighted the functional properties of MSC-EVs using *in vitro* assays and identified many factors that mediate a significant portion of the observed therapeutic effects of MSCs, including immunosuppression. Andrews et al. (37) also demonstrated that when MSCs are primed with inflammatory signals, their EVs increases compared to non-primed control MSCs.

Finally, we observed that cells cultured in the presence of 100 and 1000 μg/mL guarana presented lower PB values than the control group, which may be associated with the presence of tannins (38). Lower proliferation also occurs when MSCs are primed with IFN, a very powerful priming agent known for inducing a strong immunomodulatory phenotype with high IDO activity, PD-L1 expression, and potential to inhibit lymphocyte proliferation (39). Nevertheless, flow cytometry showed no effects in MSC cell cycle, indicating no cycle arrest in the studied concentrations. These results are interesting, since *P. cupana* can be used as a source of bioactive substances, generating cellular products with lower potential of tumorigenesis after transplantation (40).

The limitations of our study include the characterization of extracellular vesicles. Our analysis focused on quantifying and sizing the vesicles to offer insights into the content released by the cells - commonly referred to as conditioned medium - following treatments with guarana or caffeine. However, additional studies evaluating these products could provide complementary results. Therefore, this study shows *in vitro* evidence that guarana at a concentration of 1000 µg/mL could be a promising alternative for activating mesenchymal stromal cells in order to promote better cellular products for future clinical therapies, and the effect was dose-dependent. However, additional functional assays are needed to better understand the observed cellular effects.
